# Application of Computational Methods in Understanding Mutations in *Mycobacterium tuberculosis* Drug Resistance

**DOI:** 10.3389/fmolb.2021.643849

**Published:** 2021-09-28

**Authors:** Grace Mugumbate, Brilliant Nyathi, Albert Zindoga, Gadzikano Munyuki

**Affiliations:** ^1^ Department of Chemical Sciences, Midlands State University, Gweru, Zimbabwe; ^2^ Department of Chemistry, Chinhoyi University of Technology, Chinhoyi, Zimbabwe

**Keywords:** mutations, drug resistance, computational tools, *Mycobacterium tuberculosis*, molecular modeling

## Abstract

The emergence of drug-resistant strains of *Mycobacterium tuberculosis* (*Mtb*) impedes the End TB Strategy by the World Health Organization aiming for zero deaths, disease, and suffering at the hands of tuberculosis (TB). Mutations within anti-TB drug targets play a major role in conferring drug resistance within *Mtb*; hence, computational methods and tools are being used to understand the mechanisms by which they facilitate drug resistance. In this article, computational techniques such as molecular docking and molecular dynamics are applied to explore point mutations and their roles in affecting binding affinities for anti-TB drugs, often times lowering the protein’s affinity for the drug. Advances and adoption of computational techniques, chemoinformatics, and bioinformatics in molecular biosciences and resources supporting machine learning techniques are in abundance, and this has seen a spike in its use to predict mutations in *Mtb*. This article highlights the importance of molecular modeling in deducing how point mutations in proteins confer resistance through destabilizing binding sites of drugs and effectively inhibiting the drug action.

## Introduction

**FIGURE 1:**
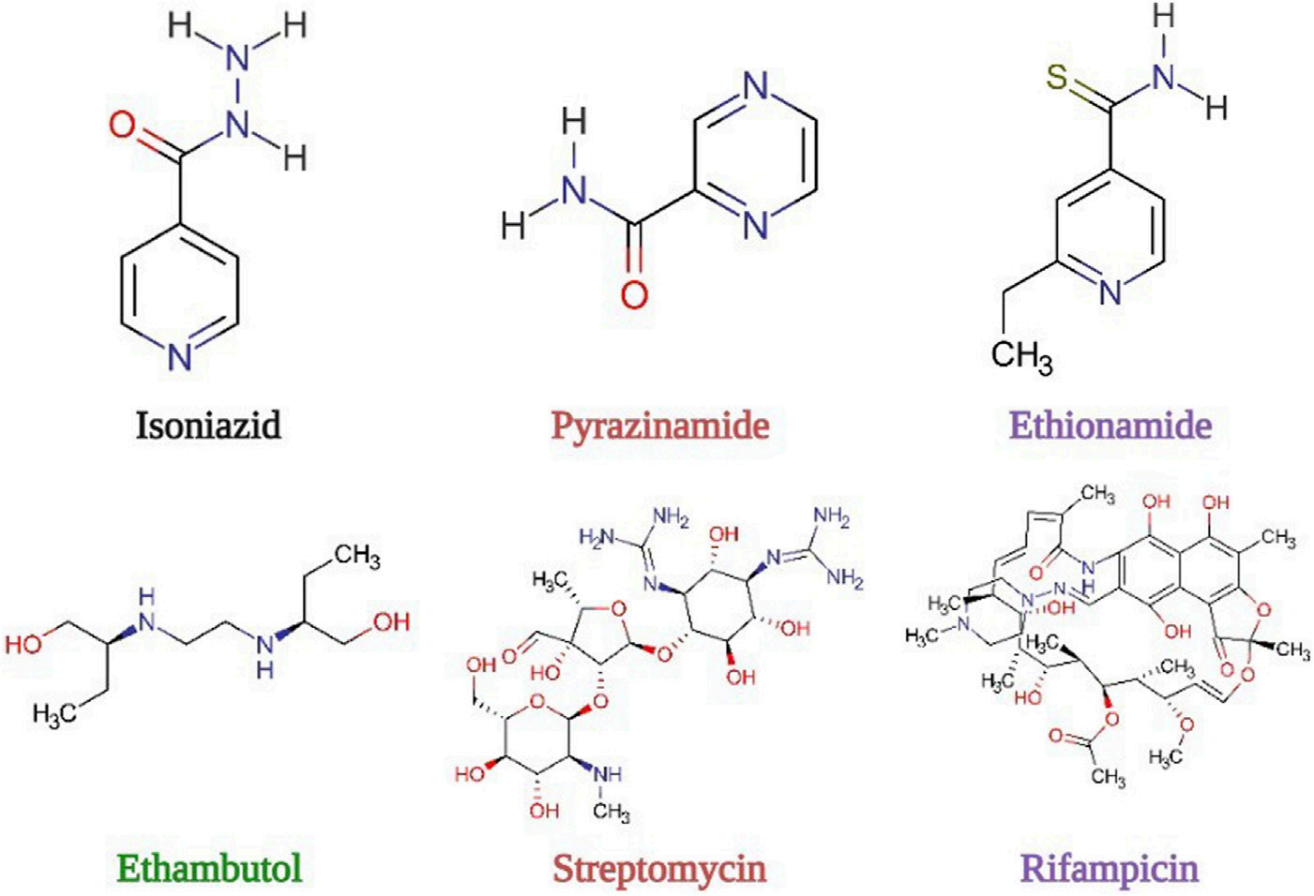
Structures of first-line drugs and ethionamide, a second-line drug.

Drug resistance in tuberculosis chemotherapy is fast becoming a health crisis on a global scale. The emergence of multidrug-resistant (MDR), extensively drug-resistant (XDR), and totally drug-resistant (TDR) strains of *Mycobacterium tuberculosis* (*Mtb*) has been observed as a result of ineffective directly observed treatment short-course (DOTS) (Bihari et al., 2008; Whalen, 2006) among a myriad of other factors. MDR is due to resistance to at least one first-line drug (Figure 1) including isoniazid (INH) which inhibits mycolic acid synthesis (Bollela et al., 2016) and rifampicin (RIF) that inhibits RNA synthesis (Zhang et al., 2019). Other TB drugs facing resistance include ethambutol (EMB) that targets the arabinogalactan synthesis (Zhang and Yew, 2009), streptomycin (STR) that inhibits protein synthesis (Ruiz et al., 2002), and pyrazinamide (PZA) that inhibits pantothenate and CoA synthesis, disrupting plasma membrane and energy metabolism (Zhang et al., 2014).

Resistance to first-line drugs leads to the implementation of treatment regiments belonging to the second-line drugs which are fluoroquinolones, kanamycin/amikacin and capreomycin/viomycin, and ethionamide whose mechanisms of action involve introducing negative supercoils in DNA molecules, inhibiting protein synthesis, and disrupting cell wall biosynthesis by inhibiting mycolic acid synthesis, respectively (Table 1). XDR and TDR are, therefore, due to resistance to several second-line drugs including fluoroquinolones in conjunction with MDR. For better management of drug resistance and rapid detection of resistance, knowledge of the mechanism of resistance at the molecular level is extremely important for an effective treatment regimen to be prescribed.

**TABLE 1:** Drug targets and the mode of action (Louw et al., 2009; Zhang and Yew, 2009).

Drug	Target	Gene	Drug mode of action
Ethambutol	Arabinosyl transferase	*embCAB*	Inhibits arabinogalactan synthesis
Streptomycin	Ribosomal protein S12	*rpsL*	Inhibits protein synthesis
	16S rRNA	*rrs*	
	7-Methylguanosine methyltransferase	*gidB*	
Pyrazinamide	Pyrazinamidase	*pncA*	Disrupts plasma membrane and energy metabolism (inhibits pantothenate and CoA synthesis)
Rifampicin	β subunit of RNA polymerase	*rpoB*	Inhibits RNA synthesis
Isoniazid	Fatty acid enoyl acyl carrier protein reductase A	*InhA*	Inhibits mycolic acid synthesis
	Catalase peroxidase	*katG*	
	β-Ketoacyl-ACP synthase	*kasA*	
	NADH dehydrogenase	*ndh*	
	Alkyl hydroperoxidase reductase	*ahpC*	
Ethionamide	Flavin monooxygenase	*ethA*	Disrupts cell wall biosynthesis by inhibition of mycolic acid synthesis
	Fatty acid enoyl acyl carrier protein reductase A	*InhA*	
	Transcriptional repressor	*ethR*	
Kanamycin/Amikacin	16S rRNA	*rrs*	Inhibits protein synthesis
Capreomycin/Viomycin	rRNA methyltransferase	*tlyA*	
	16S rRNA	*rrs*	
Fluoroquinolones	DNA gyrase	*gyrA*	Introduces negative supercoils in DNA molecules
		*gyrB*	

More often, drug resistance in *Mtb* is associated with mutations within the drug targets; however, not all mutations within the organism are associated with resistance. Drug resistance mechanisms are driven mainly by single-nucleotide polymorphisms or other polymorphisms resulting in the modification of drug targets (Palomino and Martin, 2014). Therefore, understanding the mechanism of action and resistance of the drugs is of paramount importance. Of the first-line drugs, ethambutol, which is active against fast-multiplying bacteria, disrupts the synthesis of arabinogalactan in the cell wall by targeting the *mycobacterial* arabinosyl transferase enzyme encoded by the gene *embB*, encapsulated in the embCAB operon, and mutations in the *embB*306 gene confers ethambutol resistance (Zhang and Yew, 2009). On the other hand, streptomycin, a drug active against slow-growing bacteria, irreversibly binds to the 30S ribosome subunit, blocking translation thereby inhibiting protein synthesis. Chromosomally acquired streptomycin resistance is associated with mutations in the *rpsL*, *rrs*, *and gidB* encoding for ribosomal protein S12, 16S rRNA, and 7-methylguanosine methyl transferase, respectively (Zhang and Yew, 2009). Similarly, resistance to rifampicin, a key component in the first-line treatment of TB that binds to the *β* subunit of RNA polymerase, has been linked to mutations in a region of the 81 bp region of the *rpoB* gene. Whilst the gene encodes for the *β* subunit of RNA polymerase, rifampicin resistance is mostly due to mutations at positions 516, 526, and 531 (Goldstein, 2014; Uddin et al., 2020). This is achieved by inhibition of elongation of the messenger RNA, which interferes with transcription (Uddin et al., 2020).

Pyrazinamide is also a key antituberculosis (TB) drug that substantially enhances the activity of novel agents bedaquiline (BDQ) and pretomanid (PA50) in murine models of TB. A vital attribute of this prodrug is its ability to inhibit semidormant bacteria in acidic environments. In its activity, the prodrug is converted by pyrazinamidase/nicotinamidase to its active form, pyrazinoic acid which inhibits membrane transport by disrupting the bacterial membrane energetics. Resistance to pyrazinamide is mainly characterized by mutations clustered at positions 3–17, 61–85, and 132–142 in the *pncA* gene that codes for *mycobacterial* enzyme pyrazinamidase (PZase) (Zhang et al., 2014). The association of multiple mutations throughout the *pncA* gene with PZA resistance makes it difficult to develop a test for detecting PZA resistance (Piersimoni et al., 2013). In most instances, molecular methods are applied to investigate PZA resistance by screening mutations in *pncA* genes in distinct epidemiological regions offering a much more rapid alternative method compared to that of conventional bacteriology (Khan et al., 2019). Miotto identified 280 mutations in 1950 clinical strains (Miotto et al., 2014), which were categorized into four groups: very high–confidence resistance mutations, high-confidence resistance mutations, mutations with an unclear role, and mutations not associated with phenotypic resistance based on the confidence level.

Isoniazid and ethionamide are effective drugs for the treatment of TB; however, several clinical MDR-TB strains have shown high levels of resistance (Machado et al., 2012). Structurally, INH and ETH are highly similar, both containing the pyridine ring; however, ETH is a second-line drug primarily used to treat MDR-TB, and just like INH, it is a prodrug that requires metabolic activation (DeBarber et al., 2000). Although the active metabolites of both drugs inhibit an NADH-enoyl acyl protein reductase, InhA, the drugs have independent activation pathways. The validated drug target InhA is an enzyme involved in fatty acid biosynthesis II, which is important in the bio-production of mycolic acids. These long-chain fatty acids are responsible for the unique impermeable nature of the *Mycobacterium tuberculosis* cell wall (Dover et al., 2004; Timmins and Voja, 2006).

INH is activated by the catalase-peroxidase KatG to INH-NAD and INH-NADP adducts that effectively inhibit InhA (Timmins and Voja, 2006). Resistance to INH has been attributed to mutations or deletion in the active site of the *katG* gene, which encodes the enzyme, KatG (Hameed et al., 2018), at position S315 and position 15 in the InhA promoter region. Also, mutations in *ahpC, kasA,* and *ndh* encoding for alkyl hydroperoxidase reductase, *β*-ketoacyl ACP synthase, and NADH dehydrogenase, respectively, are associated with INH resistance (Nayak et al., 2017). Cross-resistance occurs between INH and its structural analog, and ETH has been attributed to mutations in the InhA promoter.

On the contrary, ETH is activated by the enzyme EthA encoded by the gene Rv3854c to the toxic S-oxide then to 2-ethyl-4-aminopyrimidine (DeBarber et al., 2000; Baulard et al., 2000). The transcription of the FAD-containing monooxygenase, EthA, is controlled by another gene *ethR* that encodes the protein, EthR. Earlier studies of the resistance mechanism of ethionamide revealed that an increase in the amount of EthR, a member of the TetR repressors, reduces the amount of EthA and results in ethionamide resistance by *mycobacterium tuberculosis* (DeBarber et al., 2000; Baulard, 2000). Mutation studies on MDR-TB isolates revealed the presence of EthR F110L mutants implicated in resistance to ETH. The residue F110 occupies a central position in the long cylindrical and hydrophobic ligand-binding site of EthR.

Similar to INH, ethionamide (ETH) is a second-line prodrug activated by the monooxygenase encoded by the *ethA* gene. Once activated, it forms an adduct with NAD, which inhibits the enzyme enoyl-ACP reductase, thus disrupting mycolic acid synthesis. Transcription of the monooxygenase, ethA is negatively regulated by ethR; hence, allosteric inhibition of ethR would enhance activation of ETH and computer some of the mutation processes.

The advances in computational techniques and expansions in bioinformatics and chemoinformatics have brought a sigh of relief in the study of mutations and provided a rapid drug susceptibility testing important in the detection and control of MDR/XDR TB (Shinnick et al., 2005). Therefore, in this article, we analyze the effective application of computational techniques and tools in the study and understanding of molecular target mutations in conferring drug resistance to first-line drugs and also analyze how we are applying these methods to identify inhibitors that would circumvent resistance in ethR, a gene implicated in the resistance of ethionamide as well as highlight prospects in fast and cost-effective advances to understand drug resistance of antituberculosis drugs.

## Method

To give a detailed account of how the computational techniques have been applied in the study of the contributions of mutations to the emergence of drug-resistant *Mycobacterium tuberculosis*, an extensive literature search was performed. A description of the mechanisms of action of the first-line drugs rifampicin and isoniazid as well as ethionamide, a second-line drug is given. An analysis of the common computational methods used to study the mutations in relevant genes for each drug was performed. Lastly, a detailed account of the importance of F110 in ethR, a transcription regulator implicated in the resistance of ethionamide, is presented. Modeling of the proteome for mycobacteria, and identification of the hotspots and druggability of the proteins are given.

### Computational Approaches

**FIGURE 2:**
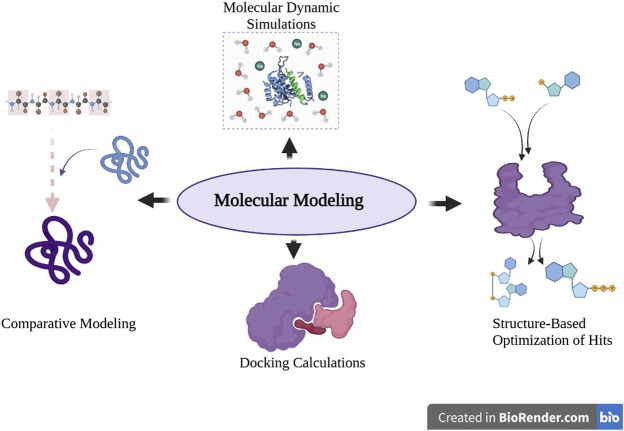
Common computational approaches applied to study the effect of mutations on protein–ligand interactions.

A variety of computational techniques that include comparative (homology) modeling, molecular dynamics, protein–ligand docking, and structure-based optimization of ligands (Figure 2) have been successfully used to study the impact of mutations at atomic levels on protein–ligand binding and interactions and how they negatively affect ligand affinity by the mutant proteins (Phelan et al., 2016; Zhang et al., 2019; Jamal et al., 2020). Advanced approaches that include machine learning alongside artificial intelligence, bioinformatics, and cheminformatics databases have also been successfully used to build models and tools that can predict mutation and determine their capabilities in conferring resistance (Jamal et al., 2020; Ghosh et al., 2020; Sandgren et al., 2009).

### Effect of Mutation in rpoB on Protein–RIF Interactions

Pang and co-workers approached RIF mutations with a computational approach. They used homology modeling to generate a three-dimensional structure of the wild type *rpoB* based on the crystal structure of *Thermus aquaticus* (Taq) core RNAP complexed with RIF. Discovery Studio 3.1 was used for this structural analysis exercise. The protein was modeled using a Build Homology module within the Protein Data Bank; a loop refinement module from Modeller was used to perform structural refinements, and energy minimizations were performed with the Smart Minimizer algorithm. The Build Mutants module was used for building mutants Ser531Leu, His526Asp, His526Gly, His526Leu, His526Arg, and Leu533Pro, and the Align and Superimpose Proteins module was used to compare the wild-type and mutant structures. Their study sought to evaluate the effects of mutating specific amino acid residues involved in the binding of RIF on protein–ligand interactions. The mutated protein–ligand interactions are evaluated subsequently using the Analyze Ligand Interactions and Structure Monitor module. They discovered that the mutated target protein had some level of resistance for RIF as it showed a decrease in its binding affinity. Mutations in His526Asp and Ser531Leu significantly reduced the affinity of *rpoB* for RIF by introducing charge repulsion and conformational changes in *rpoB*, respectively. The other strains with mutations His526Gly, His526Leu, His526Arg, and Leu533Pro exhibited low-level resistance (Pang et al., 2013). On the other hand, Zhang approached this challenge in exploring resistance mechanisms by combining the molecular dynamics simulation, molecular mechanics generalized-Born surface area calculation, dynamic network analysis, and residue interaction network analysis. Molecular dynamics simulations were all performed with the Amber14 package, and it was observed that the binding free energies of RIF with the three mutants H451D/Y/R decreased with molecular mechanics generalized-Born surface area calculations. Dynamic network analysis and residue interaction network analysis indicated increased flexibility within the binding pocket due to mutation of residue 451 which in turn weakened Q438, F439, M440, D441, and S447 residue interactions within the binding pocket. Such flexibility allowed for residues meant that a hydrogen bond to RIF was lost, thus accounting for decreased RIF binding in the mutant RNA polymerase. Changes within the binding pocket in the H451R mutant are extensive, giving too much freedom for RIF to move within the pocket (Zhang et al., 2019). Therefore, H451D/Y/R mutations increased the flexibility of the active pocket which in turn weakened the binding ability of *Mtb* RNA polymerase with RIF. Thus, the H451D/Y/R mutations weaken the interaction of the mutated residue with its adjacent residues. In similar work involving homology modeling of *rpoB* and docking calculations of RIF, Kumar and Jena have shown that two mutants S450L and H445Y exhibit low binding affinity toward the wild type *rpoB*, which has high affinity for the RIF molecule (Kumar and Jena, 2014).

Singh and co-workers investigated mutations of H451. The *Mtb rpoB* sequence was obtained from UniProt, the structure was built through comparative modeling with Modeller, and it was mutated computationally at position 451 using PyMol. GROMACS version 5.0 molecular dynamics simulation was performed on all the structures to obtain stable structures at 40ns. On the stable structures, RIF was docked onto them with AutoDock 4.2, and ligand–RIF complexes were subjected to molecular dynamics and molecular mechanics for estimation of free binding energies in wild-type and mutant systems. Resistance in the mutants arises due to changes within the binding pocket when polar and hydrophobic amino acids were replaced, which affected packing and folding in the vicinity, and relocation of the binding site itself rendering the RNA exit channel inaccessible to the drug (Singh et al., 2017).

The aforementioned studies on RIF resistance all have a consensus on the conference of resistance by the mutations in the target protein. They showed that mutations in *rpoB* cause structural changes within the binding pocket and its vicinity. They also indicated that interactions between the binding pocket residues are changed as a result of a mutation within the binding pocket and its vicinity greatly affecting the location and structure of the binding pocket. Most of these studies concluded that mutations that cause extensive structural changes will affect the way RIF sits in the binding pocket and increase freedom for the ligand in the pocket, which greatly decreases its affinity. Mutations that specifically occur within the binding pocket starve the RIF ligands of residues that contribute to a better binding affinity.

### Effect of Mutation in InhA, and katG on INH Binding

Computational studies of INH resistance in *Mtb* have been extensively studied (Jena et al., 2014). INH is activated by *katG* and converted to an active intermediate displaying antimycobacterial properties; in the presence of NADH, an INH-NAD adduct is formed. It is the adduct that inhibits *InhA* (2-trans-enoyl-acyl carrier protein reductase), blocking the synthesis of mycolic acid (Dookie et al., 2018). In one study, homology modeling was employed to predict the 3D structure of *Mtb* UDP-galactopyranose mutase (Glf) and NADH Dehydrogenase (Ndh) with Modeller9v14, and the sequence in the FASTA format was obtained from the NCBI database. The NAD binder server was used in the identification of the binding site; docking studies and visualization were performed with AutoDock Vina Tool 1.5.4 and Pymol, respectively. FADH2 and NADH were both found to have a high affinity for Glf; thus, overexpression of Glf utilizes more NADH reducing its concentration. This results in decreased INH-NAD adduct formation thereby causing INH resistance (Nayak et al., 2017)

In another study, on the influence of mutation in INH, *katG* mutations S315T/S315N were modeled with Modeller9v10 and compared with the wild-type *katG*. It was observed that INH was forming a hydrogen bond with the mutant *katG* which hindered radical formation. AutoDock Tool 1.5.4 docking calculation indicated INH-NAD is more effective at inhibiting *InhA* compared to INH (Jena et al., 2014). The *katG* mutation S315T was computationally observed to decrease the flexibility of binding site residues, and *katG* mutants at His276Met, Gln295His, and Ser315Thr decreased the stability and flexibility of the mutant protein associated with INH resistance. Mutation of the arylamine N-acetyltransferase (NAT) enzyme increases the stability and catalytic activity of the enzyme making the NAT-INH interaction ineffective. Mutations in the *ahpC* result in overexpression of the protein, which is a compensatory mechanism for loss of activity due to the *katG* mutation; thus, the ability to defend against oxidative stress is maintained within the system (Jena and Wankhade, 2016; Waghmare, and Harinath, 2016). Just as in the RIF studies, conformational changes and pocket flexibility changes greatly affect the atomic-level interactions between the target protein and the drug compound, and the trend shows a decreased affinity for the drug by mutant protein targets.

### Other Studies on the Effect of Mutation Mtb Drug Resistance

Deedler applied machine learning approaches to *Mtb* isolates that had undergone whole-genome sequencing. Nonparametric classification tree and gradient-boosted tree models were used to predict drug resistance alongside uncovering any associated new mutations. Resistance markers to drugs other than the drug of interest was used in fitting separate drug models for each drug based on the presence and absence of the co-occurrent resistance markers. Predictive performance testing was performed alongside laboratory drug-susceptibility testing. The performance was highest for resistance to first-line drugs, amikacin, kanamycin, ciprofloxacin, moxifloxacin, and multidrug-resistant tuberculosis. The inclusion of resistance markers led to improved results (Deelder et al., 2019).

In a bid to understand the molecular consequences of polymorphisms within loci associated with antituberculosis drugs, Portelli and co-workers employed computational methods to quantify point mutations in conferring resistance. Homology models of target proteins were built with UCSF Chimera 1.1, and protein–ligand docking and protein–ligand interactions were carried out with GLIDE and Arpeggio, respectively. Portelli et al., 2018 concluded that mutational effects are mostly imparted *via* steric or electrostatic changes within the protein, leading to functional changes and affecting target–drug interactions. They also noted that most phenotypically resistant mutations act allosterically, and the introduction of variants affects the drug–protein complex stability, leading to resistance. Frequently occurring mutations do not confer extreme changes in parameters; the protein retains its functionality, but the drug–protein complex is weakened. Mildly stabilizing mutations may confer local fitness advantages. Drug-resistant mutations within the protein are enhanced while maintaining stability within the protein function. It was also concluded that concurrent mutations in close topological proximity enable localized effects of the mutation, and their combination with external mutations ensures different mechanisms that lead to drug resistance.

Nakatani and Helen, 2017 predicted that the *alr* M319T mutation observed in an XDR strain of *Mtb* would likely confer resistance to D-cycloserine (DSC) as it had been noted that the acquisition of this mutation occurred with treatment of DSC suggesting that the mutation is sufficient and necessary to confer resistance. Molecular modeling of the C-8T, M319T, Y364D, and R373L mutations provided insights into how resistance is conferred upon treatment with DSC. DSC covalently binds to an *alr* cofactor pyridoxal 5′-phosphate (PLP); this act irreversibly inhibits *alr* through disruption of the *alr-*PLP covalent bond (Fenn et al., 2003). A generated model of *Mtb, alr*, and DSC highlighted the residues 319 and 364 located directly in the active site. A mutation to aspartic acid at residue 364 introduced a shorter negatively charged side chain. Such a change affects the positioning of the phosphate moiety in PLP, potentially affecting PLP orientation in the active site. The location of the residue 319 mutation could alter the interactions with 364, likely affecting DSC inhibition. *Alr* functions as a homodimer, and the R373L mutation is not located directly in the active site; however, it is close to M319 and D320 and the dimer interface. Such a mutation is most likely going to disrupt molecular interactions at the dimer interface and greatly destabilizing the DSC binding site. This study was strategic to the pharmaceutical sector in 2015 amidst a Global Drug Facility declaration of a price reduction of the DSC drug. Understanding the resistance mechanisms was important for facilitating phenotypic and genotypic drug susceptibility testing (Stop, 2015).

Malik et al. (2012) provided insights into fluoroquinolone resistance through functional genetic analyses and structural modeling techniques. Crystal structures of the N-terminal and C-terminal domains for *gyrA* and *gyrB* were superimposed on the crystal structure of the complex of *Streptococcus pneumoniae* gyrase with a DNA substrate and levofloxacin, all obtained from the Protein Data Bank using the tool Coot (Emsley and Kevin, 2004). This study highlights that *gyrB* mutations M330I, V340L, R485C, D500A, D533A, A543T, A543V, and T546M are not sufficient to confer drug resistance. N538D, E540V, and R485C + T539N mutations did confer resistance to all fluoroquinolones whilst N538K and E540D conferred resistance to moxifloxacin only, and D500H and D500N mutations conferred resistance only to levofloxacin and ofloxacin. The importance of this study was in explaining minimum inhibitory concentrations as observed in experimental work; molecular modeling explained how resistance came about to be through a 3D spatial orientation of substitute residues in the mutant proteins.

### Effect of Mutation on ethR-Ligand Binding Affinity

**FIGURE 3:**
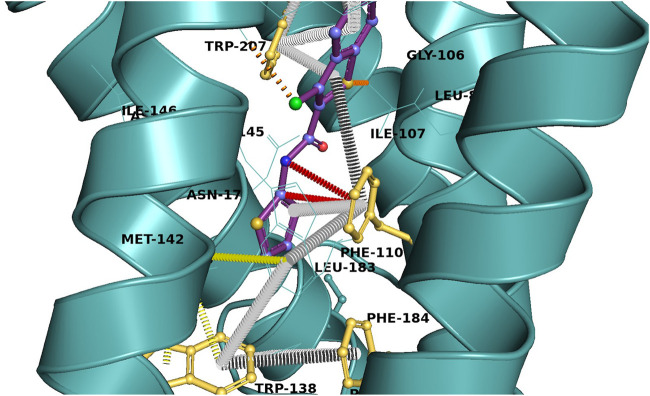
The residue F110 facilitates the pi–pi cascade (grey rings) between aromatic residues in the binding pocket of EthR (yellow) and the ligand (purple), which later translates into a structural modification of the HTH motif and inhibition of DNA binding. Analysis of the interactions was performed using Aperggio (http://bleoberis.bioc.cam.ac.uk/arpeggioweb/) and viewed using PyMol.

Resistance to ETH has been linked to mutations in the *ethR, ethA,* and *inhA* genes (Hameed et al., 2018) that collectively play crucial roles in the activity of the drug. As a regulator, the N-terminus helix-turn-helix (HTH) domains of the dimeric EthR bind DNA sequences responsible for the transcription of EthA and suppress its expression (Wolff and Nguyen, 2012). This process is controlled by small–molecular weight ligands that bind to the allosteric binding pocket of EthR located in the C-terminal end (Mugumbate et al., 2015). Binding of the ligands induces molecular conformational changes that increase the distances between DNA binding domains of the enzyme, inhibit DNA binding, and hence increase the transcription of EthA. For this reason, EthR has been validated as a suitable drug target for a new collection of antituberculosis compounds that would boost the activity of ETH. Targeting the resistance pathway of antituberculosis drugs has long been proposed (Wolff and Nguyen, 2012); therefore, independent research groups have deposited the apo and bound structures of EthR into the Protein Data Bank (PDB, https://www.rcsb.org/). These structures reveal that the protein is characterized by a long hydrophobic and promiscuous pocket that binds to structurally diverse small molecules like dioxane and long molecular chains with more than 30 atoms. The residue F110 is centrally positioned in the binding pocket with its aromatic side chain strategically positioned to participate in protein–ligand interactions (Figure 3).

In a previous study (Bishi, et al.), we carried out docking calculations of a Maybridge dataset containing more than 200 drug-like compounds to investigate binding modes and protein–ligand interactions. The results indicated that F110 played a crucial role in ligand binding, supporting the observation that F110L drastically reduces ligand affinity (Brossier et al., 2011). Most ligands were stabilized by a cascade of pi–pi interactions, where F110 played a central role by linking pi–pi interactions from the ligand to Phe114 (Figure 3) in a way that will stabilize the bound ligand and increase ligand affinity. This implies that the F110L mutation disrupts the pi–pi cascade and reduces the ligand–binding affinity.

### Application of Machine Learning and Artificial Intelligence Approaches

Bioinformatics was employed for studies concerned with mutations focusing on *Mtb*. Ghosh and co-workers developed a Drug Resistance–Associated Genes database (DRAGdb) which is a repository of mutational data of drug resistance–associated genes (DRAGs) across ESKAPE (*Enterococcus faecium, Staphylococcus aureus, Klebsiella pneumoniae, Acinetobacter baumannii, Pseudomonas aeruginosa, and Enterobacter spp*.). Homoplasy is observed in six genes namely *gidB, gyrA, gyrB, rpoB, rpsL,* and *rrs* with mutations related to drug resistance being observed at the codon level. A single-nucleotide mutation that was associated with resistance to amikacin, gentamicin, rifampicin, and vancomycin in *Staphylococcus aureus* was an indication of pleiotropy. The database compiles *Mtb* drug-resistance genes across bacterial species allowing for homoplasy and pleiotropy identification in genes (Ghosh et al., 2020).

In their recent efforts, Jamal and co-workers developed machine learning algorithms alongside artificial intelligence to study and predict resistance in the genes *rpoB, inhA, katG, pncA, gyrA,* and *gyrB* for the drugs rifampicin, isoniazid, pyrazinamide, and fluoroquinolones. Machine learning algorithms naïve Bayes, k nearest neighbor, support vector machine, and artificial neural network were used to build the prediction models. Further molecular docking and molecular dynamics simulations were carried out on predicted resistance causing mutant proteins and their wild-type counterparts. This study evaluated protein conformation and its impact to confirm the observed phenotype (Jamal et al., 2020).

## Discussion

The application of machine learning and artificial intelligence in mutation studies is a fast-growing trend in computational research. At the center of it all, bioinformatics and cheminformatics databases are contributing a lot of information that is needed by machine learning algorithms to predict drug resistance–conferring mutations. Information was gathered across species in the experimental work, where the previously mentioned mutations and their influence on drug resistance were observed in detail, and the lack of hereafter is used to train machine learning algorithms in identifying possible novel mutations that might occur and probe their potential in conferring resistance. Usage of multiple layers or algorithms (deep learning) and artificial intelligence has greatly improved the accuracy of drug resistance and mutation prediction tools that have been made available to researchers (Deelder et al., 2019; Jamal et al., 2020).

Structure-guided drug discovery has lately become paramount to combat the emergence of *Mtb* drug-resistant strains which pose a concern to global public health. The rapid expansion of genome sequencing and pathway annotations has shown a positive impact on the progress of drug discovery. Computational tools have been developed to address the effect of mutations on the structure and function of proteins. The mutation cutoff scanning matrix (mCSM) is a machine learning approach which predicts the structural and functional effects of mutations on the target proteins. Its variants are capable of predicting the effects of mutations on protein stability, protein–protein interaction, and protein–ligand interactions (Pandurangan and Blundell 2020). EnCOM and FoldX are tools that are capable of predicting the effects of mutations on flexible protein conformations (Schymkowitz et al., 2005; Frappier et al., 2015). Rapid assessment of many mutations that are difficult to access with experimental methods has been made possible through predictive learning with machine learning algorithms (Waman et al., 2019).

Machine learning techniques have also been developed to address the need to improve TB resistance prediction in less-studied drugs. Rapid detection of antimicrobial resistance is vital in the prevention of existing drug resistance amplification, given that resistance markers are known; machine learning techniques are capable of timely prediction of resistance for a given *Mtb* drug. Machine learning methods are capable of ranking mutations regarded as important and mutations from other genes associated with resistance to other drugs; this reflects on multidrug resistance from taking second-line drugs after taking first-line drugs, which is a huge advantage over experimental methods (Kouchaki et al., 2019).

DeepAMR has been developed with the task of identifying co-occurrent resistance within anti-TB drugs. This machine learning technique had a high performance with mean AUROC (Area Under the Receiver Operating Characteristics) from 94.4 to 98.7% for predicting resistance to four first-line drugs, RIF, EMB, INH, and PZA multi-drug resistant TB (MDR-TB) and pan-susceptible TB (PANS-TB: MTB that is susceptible to all four first-line anti-TB drugs). DeepAMR achieved its best mean sensitivity of 94.3, 91.5, 87.3, and 96.3% for INH, EMB, PZA, and MDR-TB, respectively. High-performance machine learning models have made the predictions of co-occurrent drug resistance to be performed timely and prevented amplification of existing resistance (Yang et al., 2019).

The use of machine learning and artificial intelligence makes them possible to identify novel resistance markers which are very difficult and costly to investigate with experimental methods. The timely and rapid prediction of drug resistance has made it possible for drugs to be returned to the discovery pipeline for optimization in a structure-guided drug design approach. To this end, the application of these techniques makes it possible for scientists to comprehensively study the protein–drug interactions at very little cost and shorter time frames.

### Proposed Computational Protocol

**FIGURE 4:**
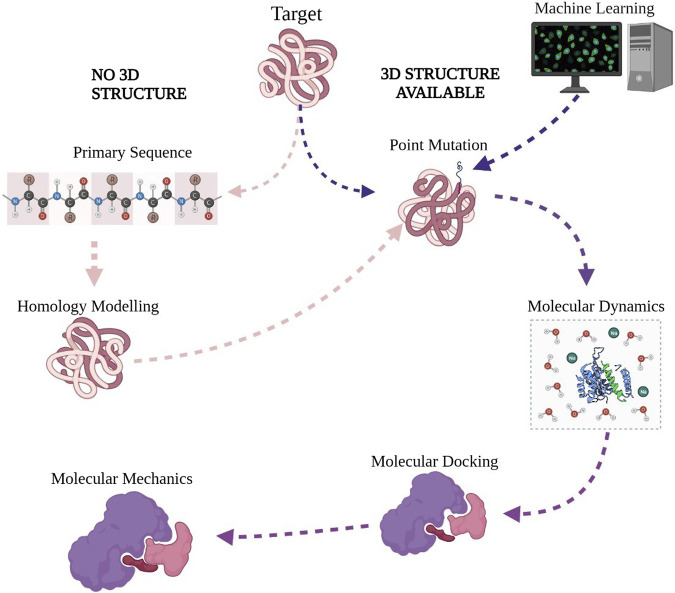
Proposed protocol for studying the effect of mutations on protein–ligand interaction.

The Application of computational tools (Table 2) in understanding mutations that confer drug resistance in *Mycobacterium tuberculosis* still require a canonization of the process for a standard result output. Initially, 3D structures of drug targets are obtained from the Protein Data Bank followed by point mutations which may be performed by changing an amino acid in a protein sequence with Pymol (Figure 4). In the absence of a 3D structure, the primary sequence of the protein is obtained from UniProt (a freely accessible database of protein sequences and functional information). 3D structures are modeled through a process known as homology/comparative modeling of proteins with a standalone program such as Modeller or, alternatively, an online server such as SWISS-MODEL (expasy.org). Molecular dynamics simulations are performed for energy minimizations of the wild-type and mutant drug targets obtaining the most stable protein structures; standalone programs such as GROMACS and Amber are used for performing the task (Singh et al., 2017; Zhang et al., 2019). In computational chemistry, energy minimizations which may also be referred to as geometry optimization entail the exploration of the conformational space for a collection of atoms to find a proper molecular arrangement in space which is energy favorable and stable; it is also referred to as the global energy minimum (Jabeen et al., 2019). The resultant structures are then subjected to molecular docking, where the position of the ligand when bound to a protein receptor is predicted for the drug’s wild type and mutated targets. AutoDockTools and Glide among other standalone software packages may be used for this task (Kumar and Jena 2014; Jamal et al., 2020). Protein–ligand complex structures may also undergo energy minimization with molecular dynamics (Kumar and Jena, 2014; Portelli et al., 2018). In the presence of a 3D structure complexed with the preferred drug, molecular mechanics is employed to probe free binding energies and compare protein–ligand complexes for wild-type and mutated drug targets (Zhang et al., 2019). Recent trends that are being explored in the field of computational work include the usage of machine learning algorithms to build prediction tools (Lee et al., 2020). Studies that make use of mathematical models alongside bioinformatics for drug resistance mutations have also been reported (Fonseca et al., 2015). There has also been an exploration of artificial intelligence alongside machine learning algorithms for drug resistance mutation predictive tests (Deelder et al., 2019).

**TABLE 2:** Computational tools used in the study of mutations.

Computational tool	Use	References
AutoDock	Molecular docking and visualization	Morris et al. (2009)
Glide	Molecular docking and visualization	Friesner and Mainz (2006)
Pymol	Molecular visualization	DeLano (2002)
Gromacs	Molecular dynamics simulations	Van Der Spoel et al. (2005)
Amber	Molecular dynamics simulations	Case et al. (2005)
Modeller	Homology or comparative modeling of protein 3D structures	Webb and Sali (2016)
Discovery Studio	Molecular visualization	Studio (2008)
Arpeggio	A web server for calculating and visualizing interatomic interactions in protein structures	Jubb et al. (2017)
UCSF Chimera	Interactive visualization and analysis of molecular structures and related data	Pettersen et al. (2004)
DeepAMR	Predicting co-occurrent resistance of *Mycobacterium tuberculosis*	Yang et al. (2019)
EnCom	Predicting the effects of mutations on flexible protein conformations	Frappier et al. (2015)
FoldX		Schymkowitz et al. (2005)

## Concluding Remarks

With the increase in the number of drug-resistant and multidrug-resistant strains of *Mtb*, a need has arisen for techniques that are rapid for extensive studies of the previously mentioned mutations. Computational methods (Figure 4) present us with the opportunity to rapidly carry out these studies in silico with outputs comparable with experimental work at high confidence at even lower costs. Such methods have been extensively employed in exploring drug resistance in rifampicin, isoniazid, and ethionamide with the findings correlating to what is observed in experimental work; structural changes within the mutant protein drastically reduce protein–ligand binding affinity.

Machine learning and artificial intelligence have brought about massive changes and advancements in studying mutations and drug resistance in *Mtb* and other diseases. These techniques have made it possible to identify resistance markers within the whole genome Muzondiwa et al., 2020, to predict drug resistance for a given molecule, and to predict co-occurrent drug resistance between two or more drugs. The techniques are driven by big data (Table 3), and to that effect, smaller specific repositories/databases (Drug Resistance–Associated Genes database) have been created for the sole purpose of helping researchers who are studying mutations. Computational tools have also been created for the identification of resistance markers and prediction of drug resistance (DeepAMR). Predictive learning makes it possible for scientists to identify potentially unwanted drug characteristics that may not be picked up with experimental methods, greatly reducing the risk for drug failure and saving time and money in the process.

**TABLE 3:** Databases used alongside computational packages.

Database	Information contained	References
UniProt	Protein sequence and functional information	Consortium (2015)
Protein Databank	Protein 3D Structures	Berman et al. (2000)
DRAGdb	Mutational data of drug resistance–associated genes	Ghosh et al. (2020)
NCBI	Biological data and small-molecule database	Wheeler et al. (2006)
ChEMBL	Binding, functional, and ADMET information for a large number of drug-like bioactive compounds	Gaulton and LouisaBellis (2012)

## References

[B63] Baulard A. R. Joanna C. B. Jean E.-N. Selwyn Q. Ruth A. M. Patrick J. B. (2000). Activation of the pro-drug ethionamide is regulated in mycobacteria. J. Biol. Chem. 275 (36), 28326–28331. 10.1074/jbc.M003744200 10869356

[B1] Berman H. M. Westbrook J. Feng Z. Gilliland G. Bhat T. N. Weissig H. (2000). The Protein Data Bank. Nucleic Acids Res. 28 (1), 235–242. 10.1093/nar/28.1.235 10592235 PMC102472

[B2] Bihari S. Arun S. Rawat T. Katiyar S. (2008). An Analysis of Failure of Category II DOTS Therapy. Indian J. Community Med. 33 (2), 129. 10.4103/0970-0218.40886 19967042 PMC2784623

[B67] Brossier F. Veziris N. Truffot-Pernot C. Jarlier V. Sougakoff W. (2011). Molecular investigation of resistance to the antituberculous drug ethionamide in multidrug-resistant clinical isolates of Mycobacterium tuberculosis. Antimicrob. Agents Chemother. 55 (1), 355. 10.1128/AAC.01030-10 20974869 PMC3019671

[B3] Bollela V. R. Namburete E. I. Feliciano C. S. Macheque D. Harrison L. H. Caminero J. A. (2016). Detection of KatG and InhA Mutations to Guide Isoniazid and Ethionamide Use for Drug-Resistant Tuberculosis. Int. J Tuberc. Lung Dis. 20 (8), 1099–1104. 10.5588/ijtld.15.0864 27393546 PMC5310937

[B4] Case D. A. Cheatham T. E. III Darden T. Gohlke H. Luo R. Merz K. M. (2005). The Amber Biomolecular Simulation Programs. J. Comput. Chem. 26 (16), 1668–1688. 10.1002/jcc.20290 16200636 PMC1989667

[B5] Consortium U. P. (2015). UniProt: a Hub for Protein Information. Nucleic Acids Res. 43, D204–D212. 10.1093/nar/gku989 25348405 PMC4384041

[B6] DeBarber A. E. Mdluli K. Bosman M. Bekker L.-G. Barry C. E. (2000). Ethionamide Activation and Sensitivity in Multidrug-Resistant Mycobacterium tuberculosis. Proc. Natl. Acad. Sci. 97 (17), 9677–9682. 10.1073/pnas.97.17.9677 10944230 PMC16924

[B7] Deelder W. Christakoudi S. Phelan J. Benavente E. D. Campino S. McNerney R. (2019). Machine Learning Predicts Accurately Mycobacterium Tuberculosis Drug Resistance from Whole Genome Sequencing Data. Front. Genet. 10 (SEP), 1–9. 10.3389/fgene.2019.00922 31616478 PMC6775242

[B8] DeLano W. L. (2002). Pymol: An Open-Source Molecular Graphics Tool. CCP4 Newsl. Protein Crystallogr. 40 (1), 82–92.

[B9] Dookie N. Rambaran S. Padayatchi N. Mahomed S. Naidoo K. (2018). Evolution of Drug Resistance in Mycobacterium Tuberculosis: A Review on the Molecular Determinants of Resistance and Implications for Personalized Care. J. Antimicrob. Chemother. 73 (5), 1138–1151. 10.1093/jac/dkx506 29360989 PMC5909630

[B10] Dover L. G. Cerdeño-Tárraga A. M. Pallen M. J. Parkhill J. Besra G. S. (2004). Comparative Cell wall Core Biosynthesis in the Mycolated Pathogens,Mycobacterium tuberculosisandCorynebacterium Diphtheriae. FEMS Microbiol. Rev. 28 (2), 225–250. 10.1016/j.femsre.2003.10.001 15109786

[B11] Emsley P. Cowtan K. (2004). Coot: Model-Building Tools for Molecular Graphics. Acta Crystallogr. D Biol. Cryst. 60 (12), 2126–2132. 10.1107/S0907444904019158 15572765

[B12] Fenn T. D. Stamper G. F. Morollo A. A. Ringe. D. (2003). A Side Reaction of Alanine Racemase: Transamination of Cycloserine. Biochemistry 42 (19), 5775–5783. 10.1021/bi027022d 12741835

[B13] Fonseca J. D. Knight G. M. McHugh T. D. (2015). The Complex Evolution of Antibiotic Resistance in Mycobacterium Tuberculosis. Int. J. Infect. Dis. 32, 94–100. 10.1016/j.ijid.2015.01.014 25809763

[B14] Frappier V. Chartier M. Najmanovich R. J. (2015). ENCoM Server: Exploring Protein Conformational Space and the Effect of Mutations on Protein Function and Stability. Nucleic Acids Res. 43, W395–W400. 10.1093/nar/gkv343 25883149 PMC4489264

[B15] Friesner R. A. Murphy R. B. Repasky M. P. Frye L. L. Greenwood J. R. Halgren T. A. (2006). Extra Precision Glide: Docking and Scoring Incorporating a Model of Hydrophobic Enclosure for Protein−Ligand Complexes. J. Med. Chem. 49 (21), 6177–6196. 10.1021/jm051256o 17034125

[B16] Gaulton A. BellisBellis L. J. A. Bento A. P. Chambers J. Davies M. Hersey A. (2012). ChEMBL: a Large-Scale Bioactivity Database for Drug Discovery. Nucleic Acids Res. 40, D1100–D1107. 10.1093/nar/gkr777 21948594 PMC3245175

[B17] Ghosh A. N. S. Saha. S. (2020). Survey of Drug Resistance Associated Gene Mutations in Mycobacterium Tuberculosis, ESKAPE and Other Bacterial Species. Sci. Rep. 10 (1), 1–11. 10.1038/s41598-020-65766-8 32488120 PMC7265455

[B18] Goldstein B. P. (2014). Resistance to Rifampicin: A Review. J. Antibiot. 67, 625–630. Nature Publishing Group. 10.1038/ja.2014.107 25118103

[B19] Hameed H. M. A. Islam M. M. Chhotaray C. Wang C. Liu Y. Tan Y. (2018). Molecular Targets Related Drug Resistance Mechanisms in MDR-, XDR-, and TDR-Mycobacterium Tuberculosis Strains. Front. Cel. Infect. Microbiol. 8, 114. 10.3389/fcimb.2018.00114 PMC5932416 29755957

[B20] Jabeen A. Mohamedali A. Ranganathan S. (2019). “Protocol for Protein Structure Modelling,” in Encyclopedia of Bioinformatics and Computational Biology. Oxford: Academic, 252–272. 10.1016/B978-0-12-809633-8.20477-9

[B21] Jamal S. Khubaib M. Gangwar R. Grover S. Grover A. Hasnain S. E. (2020). Artificial Intelligence and Machine Learning Based Prediction of Resistant and Susceptible Mutations in Mycobacterium Tuberculosis. Sci. Rep. 10 (1), 1–16. 10.1038/s41598-020-62368-2 32218465 PMC7099008

[B22] Jena L. Waghmare P. Kashikar S. Kumar S. Harinath B. C. (2014). Computational Approach to Understanding the Mechanism of Action of Isoniazid, an Anti-TB Drug. Int. J. Mycobacteriology 3 (4), 276–282. 10.1016/j.ijmyco.2014.08.003 26786627

[B23] Jubb H. C. Higueruelo A. P. Ochoa-Montaño B. Pitt W. R. Ascher D. B. Blundell T. L. (2017). Arpeggio: a Web Server for Calculating and Visualising Interatomic Interactions in Protein Structures. J. Mol. Biol. 429 (3), 365–371. 10.1016/j.jmb.2016.12.004 27964945 PMC5282402

[B24] Khan M. T. Malik S. I. Ali S. Masood N. Nadeem T. Khan A. S. (2019). Pyrazinamide Resistance and Mutations in PncA Among Isolates of Mycobacterium Tuberculosis from Khyber Pakhtunkhwa, Pakistan. BMC Infect. Dis. 19 (1), 1–7. 10.1186/s12879-019-3764-2 30728001 PMC6364397

[B25] Kouchaki S. Yang Y. Walker T. M. Sarah Walker A. Wilson D. J. Peto T. E. A. (2019). Application of Machine Learning Techniques to Tuberculosis Drug Resistance Analysis. Bioinformatics 35 (13), 2276–2282. 10.1093/bioinformatics/bty949 30462147 PMC6596891

[B26] Kumar S. Jena L. (2014). Understanding Rifampicin Resistance in Tuberculosis through a Computational Approach. Genomics Inform. 12 (4), 276. 10.5808/gi.2014.12.4.276 25705170 PMC4330266

[B27] L J. G W. (2016). Computational Approach in Understanding Mechanism of Action of Isoniazid and Drug Resistance. Mycobact Dis. 06 (01), 1–3. 10.4172/2161-1068.1000202

[B28] Lee B. M. Harold L. K. Almeida D. V. Aung H. L. Forde B. M. Hards K. (2020). Predicting Nitroimidazole Antibiotic Resistance Mutations in Mycobacterium Tuberculosis with Protein Engineering. Plos Pathog. 16 (2), e1008287. 10.1371/journal.ppat.1008287 32032366 PMC7032734

[B29] Louw G. E. Warren R. M. Gey Van Pittius N. C. McEvoy C. R. E. Van Helden P. D. Victor T. C. (2009). A Balancing Act: Efflux/Influx in Mycobacterial Drug Resistance. Antimicrob. Agents Chemother. 53 (8), 3181–3189. 10.1128/AAC.01577-08 19451293 PMC2715638

[B64] Machado D. Isabel C. B. João P. Liliana P. Pedro B. Isabel J. B. Bruno V. (2012). Contribution of efflux to the emergence of isoniazid and multidrug resistance in Mycobacterium tuberculosis. PLoS One 7 (4), e34538. 10.1371/journal.pone.0034538 22493700 PMC3321020

[B30] Malik S. Willby M. Sikes D. Tsodikov O. V. Posey J. E. (2012). New Insights into Fluoroquinolone Resistance in *Mycobacterium tuberculosis*: Functional Genetic Analysis of gyrA and gyrB Mutations. PloS one 7 (6), e39754. 10.1371/journal.pone.0039754 22761889 PMC3386181

[B31] Miotto P. Cabibbe A. M. Feuerriegel S. Casali N. Drobniewski F. Rodionova Y. (2014). *Mycobacterium tuberculosis* Pyrazinamide Resistance Determinants: a Multicenter Study. MBio 5 (5), e01819. 10.1128/mBio.01819-14 25336456 PMC4212837

[B32] Morris G. M. Huey R. Lindstrom W. Sanner M. F. Belew R. K. Goodsell D. S. (2009). AutoDock4 and AutoDockTools4: Automated Docking with Selective Receptor Flexibility. J. Comput. Chem. 30 (16), 2785–2791. 10.1002/jcc.21256 19399780 PMC2760638

[B33] Mugumbate G. Abrahams K. A. Cox J. A. G. Papadatos G. van Westen G. Lelièvre J. (2015). Mycobacterial Dihydrofolate Reductase Inhibitors Identified Using Chemogenomic Methods and *In Vitro* Validation. PloS one 10, 3e0121492. 10.1371/journal.pone.0121492 PMC4370846 25799414

[B34] Muzondiwa D. Mutshembele A. Pierneef R. E. Reva O. N. (2020). Resistance Sniffer: an Online Tool for Prediction of Drug Resistance Patterns of *Mycobacterium tuberculosis* Isolates Using Next Generation Sequencing Data. Int. J. Med. Microbiol. 310 (2), 151399. 10.1016/j.ijmm.2020.151399 31980371

[B35] Nakatani Y. Opel-Reading H. K. Merker M. Machado D. Andres S. Kumar S. S. (2017). Role of Alanine Racemase Mutations in *Mycobacterium tuberculosis* D -Cycloserine Resistance. Antimicrob. Agents Chemother. 61, e01575–17. 10.1128/AAC.01575-17 28971867 PMC5700341

[B36] Nayak T. Jena L. Bc H. (2017). “Austin Tuberculosis : Research & Treatment Isoniazid Drug Resistance : Computational Study to Understand the Mechanism of over Expressed UDP- Galactopyranose Mutase Enzyme in Causing Drug Resistance in Tuberculosis. Austin Tuberculosis: Res. Treat. 2 (1), 2–7. Available at: https://austinpublishinggroup.com/tuberculosis/fulltext/atrt-v2-id1006.php . 10.4103/ijmy.ijmy_174_17

[B37] Palomino J. C. Martin A. (2014). Drug Resistance Mechanisms in Mycobacterium Tuberculosis. Antibiotics 3 (3), 317–340. 10.3390/antibiotics3030317 27025748 PMC4790366

[B38] Pandurangan A. P. Blundell T. L. (2020). Prediction of Impacts of Mutations on Protein Structure and Interactions: SDM, a Statistical Approach, and mCSM, Using Machine Learning. Protein Sci. 29 (1), 247–257. 10.1002/pro.3774 31693276 PMC6933854

[B39] Pang Y. Lu J. Wang Y. Song Y. Wang S. Zhao Y. (2013). Study of the Rifampin Monoresistance Mechanism in *Mycobacterium tuberculosis* . Antimicrob. Agents Chemother. 57 (2), 893–900. 10.1128/AAC.01024-12 23208715 PMC3553728

[B40] Pettersen E. F. Goddard T. D. Huang C. C. Couch G. S. Greenblatt D. M. Meng E. C. (2004). UCSF Chimera? A Visualization System for Exploratory Research and Analysis. J. Comput. Chem. 25 (13), 1605–1612. 10.1002/jcc.20084 15264254

[B41] Phelan J. Coll F. McNerney R. Ascher D. B. Pires D. E. V. Furnham N. (2016). *Mycobacterium tuberculosis* Whole Genome Sequencing and Protein Structure Modelling Provides Insights into Anti-tuberculosis Drug Resistance. BMC Med. 14 (1), 1–13. 10.1186/s12916-016-0575-9 27005572 PMC4804620

[B42] Piersimoni C. Mustazzolu A. Giannoni F. Bornigia S. Gherardi G. Fattorini L. (2013). Prevention of False Resistance Results Obtained in Testing the Susceptibility of Mycobacterium Tuberculosis to Pyrazinamide with the Bactec MGIT 960 System Using a Reduced Inoculum. J. Clin. Microbiol. 51 (1), 291–294. 10.1128/JCM.01838-12 23100351 PMC3536208

[B43] Portelli S. Phelan J. E. Ascher D. B. Clark T. G. Furnham N. (2018). Understanding Molecular Consequences of Putative Drug Resistant Mutations in Mycobacterium Tuberculosis. Sci. Rep. 8 (1), 1–12. 10.1038/s41598-018-33370-6 30337649 PMC6193939

[B44] Ruiz P. Rodríguez-Cano F. Zerolo F. J. Casal M. (2002). Investigation of the In Vitro Activity of Streptomycin Against Mycobacterium Tuberculosis. Microb. Drug Resist. 8 (2), 147–149. 10.1089/107662902760190707 12118520

[B65] Sandgren A. Michael S. Preetika M. Brian K. W. George M. C. Megan B. M. (2009). Tuberculosis drug resistance mutation database. PLoS Med. 6 (2), e1000002. 10.1371/journal.pmed.1000002 PMC2637921 19209951

[B45] Schymkowitz J. Borg J. Stricher F. Nys R. Rousseau F. Serrano L. (2005). The FoldX Web Server: an Online Force Field. Nucleic Acids Res. 33, W382–W388. 10.1093/nar/gki387 15980494 PMC1160148

[B46] Shinnick T. M. Iademarco M. F. Ridderhof J. C. (2005). National Plan for Reliable Tuberculosis Laboratory Services Using a Systems Approach. Recommendations from CDC and the Association of Public Health Laboratories Task Force on Tuberculosis Laboratory Services. MMWR. Recomm. Rep: Morbidity Mortality Weekly Rep. 54 (RR-6), 1–12. Recommendations and Reports/Centers for Disease Control. Available at: http://europepmc.org/abstract/MED/15829862 . 15829862

[B47] Singh A. Grover S. Sinha S. Das M. Somvanshi P. Grover A. (2017). Mechanistic Principles behind Molecular Mechanism of Rifampicin Resistance in Mutant RNA Polymerase Beta Subunit of Mycobacterium Tuberculosis. J. Cel. Biochem. 118 (12), 4594–4606. 10.1002/jcb.26124 28485504

[B48] Stop T. B. (2015). Stop TB Partnership’s Global Drug Facility (GDF) Achieves Historic price Reduction for MDR-TB Drug Cycloserine. Geneva, Switzerland: Stop TB Partnership.

[B49] Studio, Discovery (2008). Dassault systemes BIOVIA, Discovery studio modelling environment, Release 4.5. Accelrys Softw Inc, 98–104. 10.4016/8372.01

[B50] Timmins G. S. Deretic V. (2006). Mechanisms of Action of Isoniazid. Mol. Microbiol. 62 (5), 1220–1227. 10.1111/j.1365-2958.2006.05467.x 17074073

[B51] Uddin M. K. M. Rahman A. Ather M. F. Ahmed T. Rahman S. M. M. Ahmed S. (2020). Distribution and Frequency of rpoB Mutations Detected by Xpert MTB/RIF Assay Among Beijing and Non-Beijing Rifampicin Resistant *Mycobacterium tuberculosis* Isolates in Bangladesh. Idr 13, 789–797. 10.2147/IDR.S240408 PMC7073589 32210593

[B52] Van Der Spoel D. Lindahl E. Hess B. Groenhof G. Mark A. E. Berendsen H. J. C. (2005). GROMACS: Fast, Flexible, and Free. J. Comput. Chem. 26 (16), 1701–1718. 10.1002/jcc.20291 16211538

[B53] Waman V. P. Vedithi S. C. Thomas S. E. Bannerman B. P. Munir A. Skwark M. J. (2019). Mycobacterial Genomics and Structural Bioinformatics: Opportunities and Challenges in Drug Discovery. Emerg. Microbes.Infect. 8, 109–118. 10.1080/22221751.2018.1561158 30866765 PMC6334779

[B54] Webb B. Sali A. (2016). Comparative Protein Structure Modeling Using MODELLER. Curr. Protoc. Bioinformatics 54 (1), 5–6. 10.1002/cpbi.3 27322406 PMC5031415

[B55] Whalen C. C. (2006). Failure of Directly Observed Treatment for Tuberculosis in Africa: A Call for New Approaches. Clin. Infect. Dis. 42 (7), 1048–1050. 10.1086/501022 16511774

[B56] Wheeler D. L. Barrett T. Benson D. A. Bryant S. H. Canese K. Chetvernin V. (2006). Database Resources of the National center for Biotechnology Information. Nucleic Acids Res. 34, D173–D180. 10.1093/nar/gkj158 16381840 PMC1347520

[B57] Wolff K. A. Nguyen. L. (2012). Strategies for Potentiation of Ethionamide and Folate Antagonists against Mycobacterium Tuberculosis. Expert Rev. anti-infective Ther. 10, 971–981. 10.1586/eri.12.87 PMC3971469 23106273

[B58] Yang Y. Walker T. M. Walker A. S. Wilson D. J. Peto T. E. A. Crook D. W. (2019). DeepAMR for Predicting Co-occurrent Resistance of *Mycobacterium tuberculosis* . Bioinformatics 35, 3240–32493249. 10.1093/bioinformatics/btz067 30689732 PMC6748723

[B60] Zhang Q. An X. Liu H. Wang S. Xiao T. Liu H. (2019). Uncovering the Resistance Mechanism of Mycobacterium Tuberculosis to Rifampicin Due to RNA Polymerase H451D/Y/R Mutations from Computational Perspective. Front. Chem. 7 (December), 1–13. 10.3389/fchem.2019.00819 31850310 PMC6902089

[B61] Zhang Y. Shi W. Zhang W. Mitchison D. (2014). Mechanisms of Pyrazinamide Action and Resistance. Microbiol. Spectr. 2 (4), 1–12. 10.1128/microbiolspec.mgm2-0023-2013 PMC4268777 25530919

[B62] Zhang Y. Yew W.-W. (2015). Mechanisms of Drug Resistance in *Mycobacterium tuberculosis*: Update 2015. Int. J. Tuberculosis Lung Dis. 19, 1276–1289. 10.5588/ijtld.15.0389 26467578

